# Mechanical Dentistry

**Published:** 1870-05

**Authors:** J. Z. Hoffer


### ARTICLE II.

### *Mechanical Dentistry.*

By J. Z. Hoffer, D. D. S.

Taking an impression of the mouth may be considered a simple operation, yet one in my judgement requiring care and skill in order to secure good results, it being the foundation upon which we are to erect our superstructure; for unless we secure a correct impression of the part to be covered by plate, our artificial denture, no matter however skillfully made, and artistically finished, will be worthless for any practical purpose in the mouth. Admitting this to be the case, it is of the greatest importance that a correct beginning should be made. In order to secure good results, the first thing to be considered is the condition of the mouth; if a hard smooth gum exists, or a soft spongy movable one; if a hard or soft palate; if any arch worth of the name, or one running off at an angle of forty-five degrees; in a word, to fully understand the difficulties, if any exist. This observation also determines the material to be used in taking the impression, whether plaster or wax, these being the only substances which can be used with success. The peculiarities of the mouth fully understood and the substance decided upon, the next thing to be considered is the selection of a proper vessel or cup in which to take the impression; this should be in a measure adapted to the mouth, so that no more material need be used than is really necessary, which will avoid crowding the mouth and causing the unpleasantness of an excess of material extending into the fauces; it also lessens the liability to alter, if wax is used in removing it as in the case of teeth remaining or, as is occasionally found teeth upon one side only. The price of the cup should not be considered, but it be rendered adaptable; our motto being "start right and endeavor to keep so." The cup and material ready—say wax, and the impression an upper one, the patient should be seated in an ordinary chair, the wax evenly softened, and adjusted in the cup as near the shape from observation of the mouth as is attainable by moulding with the fingers. Then holding the cup between the finger and thumb of the one hand, we take our stand at the side of the patient, and with the fore finger of the other press the lip and cheek out of the way and introduce the cup, placing it fairly over the ridge; then with the two first fingers of each hand placed in the bottom of cup, the thumbs resting on the handle to steady it, make firm even pressure until it is brought to the position desired. Holding the cup in place, let the thumbs complete it by pressing the wax well in to the alveolar border; allowing it time to stiffen; then remove it carefully by taking the handle of the cup again between the index finger and thumb, and with the index finger of the other hand raise the cheek so as to put some strain on the muocus membrane covering the ridge, at the same time making gentle pressure on the cup downwards; this will, by admitting air, loosen the impression which should be removed with care. If a plaster impression of the mouth is desired, take the wax impression while it is yet soft and press that portion covering the alveolar border out so as to enlarge the impression a trifle, and with the point of a knife blade prick the whole surface so that the plaster may have more hold on the wax; then with the knife slightly warmed trim off all excess around the border and that portion extending back upon the soft palate. To mix plaster use a weak solution of the sulphate of potash—about one part of the saturated solution to seven of water and of the consistency of cream, pour it quickly about the eighth of an inch thick over the wax impression; then taking a teaspoonful on the point of a spatula, place this in the vault of the mouth, rubbing well over the rugæ with the finger, after which introduce the cup as elsewhere described, with the exception of bringing up the front a trifle in advance of the heel to prevent air cells. Then let it remain until the plaster will break with a decided fracture, using the same precaution in removing as given for wax. An impression taken in this way, it is believed, will give uniformly better results than where plaster is used alone, the expansion must be less where these is less bulk, and in very deep arches it is more certain to reach all points. What has been said relates of course to upper impressions alone—partial and whole; thesame general rules, however, will apply to the lower, both as to adjustment of cups and materials. Having now obtained a perfect impression, it only remains to prepare it for the reception of tbe plaster which is to constitute the model, this is done by giving the face a thin coat of varnish—I use shellac as evenly put on as possible—and when dry, oil the surface, carefully wiping off all the excess with the brush, when a batter of plaster is mixed and poured into it. While this is being done the impression should be jarred so as to force out all bubbles, which will prevent air cells, making the casts very shallow for reasons given hereafter. The impression when full is inverted upon some plaster previously put upon a piece of glass; and if the case is for a rubber base, I usually extend the cast back so as to afford a base to articulate upon. After the plaster has set, the wax after warming slightly can be pulled off, leaving the thin coat of plaster remain, which will readily scale off by gently tapping over the ridge; this gives us in a rough state the *Model.* As we refer to none of the patented pieces, but an old fashioned swaged base, we shall proceed to prepare this model for making the dies, which is done by shaping it with a knife, so as to slightly taper it from the alveolar border and back part to the base of cast. If for a suction plate, I usually, when the thin coat of plaster admits, prefer cutting the chamber in the impression, but as this can not always be done, as in the case of wax, I take a piece of wax plate slightly warmed and press upon the model, cutting out the size and shape of cavity desired, and pouring in plaster; this, if the cast is moistened, will unite firmly, doing away with the risk of falling off, when the plaster has set; I then finish off and remove the wax. If for a rimmed plate, I build wax or plaster on the outside of the model to where it is desired the the plate should terminate on the border, and with a knife shape it to any angle desired, and then varnish, and the cast is ready for use. But should the ridge present an acute angle, as in the case of a model prepared for stamping the rim, rendering it difficult to mould, plaster should be run around the border previously oiled up even with the ridge, leaving small points extending up to designate its place in the sand, when it will be found to draw from the matrix without any trouble or injury to the impression. These sections are to be replaced when the precaution is taken to drive off all moisture. The model completed, we now come, in the third place, to consider the manner of constructing and procuring *Metallic Dies.*

Various methods having been adopted, such as first casting dies direct from the impression; second, pouring melted metal in an impression made in sand with the model, and third, immersing the plaster model in molten metal until chilled, being the principal ones, and there being a diversity of opinion existing as to the merits and demerits of each particular plan—all thinking their own the best. I shall, therefore, for brevity sake confine myself to the one long ago adopted, the old fashioned one of moulding in sand, and which uniformly produces as good results as any. The materials and appliances necessary are flasks—sand sieve—ladles—spatula—zinc—tin and lead; the flasks used are the conical shaped *rings* designed by Prof. Austen, five in a nest so arranged as to form a curve when set one on top of the other; these are all that could be desired both as to economy and general application. The materials at hand and the sand dampened suficiently to remain in shape when compressed by the hand, the shallow plaster cast is placed on a level surface bottom down, and the large ring inverted over it, when sand is sieved and packed around and over the model, squaring off the sand even with the flask and with the spatula pare away the sand until merely the portion desired is enclosed; tap gently a few times on the cast and hold it in an inverted position so that when it falls out the surface may not be injured; a tap or two on the bottom will complete its removal. We have now a shallow sand impression in which the melted zinc is poured until even with the sand, when the ring next in size is placed fairly on, and pour in the balance. This should be sufficient to give a depth of two inches from the arch; and in pouring the zinc care should be used so that the metal runs around the ridge rising up over the arch, otherwise we may have displacement of the sand and boiling, especially where the plaster sections are used; also in the melting of the zinc, attention should be given so that it is not oxidized but taken just when melted, as this is of great importance in the procuring of perfect dies. After the casting has cooled, part the flask, remove the sand and brush off the casting and coat it with a solution of whiting and alcohol, and again put together the flask with the face of the die up, when it is ready to receive the counter dies, these being made of lead or tin or both combined in certain proportions; for my part I generally make from two to three dies and a counter on each, reserving the best die and tin counter for finishing. This number enables me to swage a plate adapted to all the little inequalities of the mouth; whereas when but one set is used the surface becomes so battered that it can be but a mere approximation. It is the habit of many to trust this part and the getting up of the plate to inexperienced persons, thinking it of little consequence; but it may prove labor lost.

*Swaging Plates.* The dies and counter dies being ready for use, the size and shape of plate may be obtaned by lightly swaging a piece of pattern tin foil between the dies and with the point of a knife cutting it to the required shape; then restore it to a smooth surface with the burnisher; then lay the pattern on the plate, and with a pointed instrument mark out the form and with the shears, saw and files cut out the plate, when it is annealed and then oiled to prevent any metal from adhering to it. With a hard wooden mallet bring the centre of the plate into as good an adaptation to the die as is possible before bringing the edge over the ridge: after this partial adjustment and fitting to the die, wipe off all the oil and anneal the plate and again swage between the die and counter die, repeating the operation and annealing the plate each time until a perfect adaptation is obtained ; reserving the best die and tin counter until the plate is trimmed the exact size when a final swage may be given and the plate again annealed, to see that no change has resulted from the application of heat. This is an important precaution, for unless the plate after heating retains its adaptation to the die, it is likely in soldering to warp, when it is too late to remedy it. The plate should now be cleansed when it ready to try in the mouth and obtain the bite.

*Articulating Model.* Under this head we shall include the selection of the teeth, taking the bite and making the articulating model. As the choosing of the teeth is of vast importance to the success of a piece, the dentist should exercise the greatest care in their selection not only as to the color but size and shape, also in view of their arrangement while the patient is present, ascertain to if possible what would prove best to restore harmony and a natural expression. I may here say that at every step in the process of making an artificial denture, we find that mechanical skill—although essential, is not the only qualification or requisite of a dentist, but he should possess ability to combine science with mechanic art. It is important too that he should possess some originality and design and should have made the natural teeth a study in connection with expression and features. The idea that any one, no matter how good a mechanic, can without these essentials engage succssfully in the practice of dentistry is a grave error. The plate having been found adapted to the mouth, a rim of wax is placed on the ridge or border as far as the plate extends and trimmed to represent the fullness and length of the teeth ; this is arrived at by frequently trying in the mouth, cutting off or adding thereto as necessary, until desired fullness and length is attained, when a strip of warm wax—sufficient to take impression of the tips of lower teeth, is laid on the rim and the patient directed to bite through, when a knife is used to mark the median line. If for a full denture, the upper may be trimmed as just described and the lower should be placed in the mouth and trimmed off until it gives a natural appearance. Then getting the patient to open and close the mouth as in the act of eating, catch the most natural approxmation and mark the median line; this done remove the lower and half imbed a few bird shot in the rim of wax, transferring it again to the mouth and directing the patient to close, thus imbeding the remaining half in the upper wax. The shot serve a double purpose that of a guide, and if the wax is not trimmed correctly, keeps the plate against the ridge by the resistance offered by the wax rims. They may now be removed and joined together ready for the model, which is made by pouring a batter of plaster in the upper side of plate, as described in making plaster casts, and extending back for a base to articulate upon. After the plaster has set, the portion extending behind the plate should have grooves cut to antagonize with; this burnished, and when dry oiled to prevent the plaster from adhering. The whole is then surrounded with paper or putty, and the remaining half poured, after which the model is trimmed and the wax rims removed, when the model is ready for adjusting the teeth.

*Arranging the Teeth &c.* The teeth having been selected for the case—an upper one—we proceed to adjust the central incisors; in arranging these and more particularly the laterals we should remember the patients peculiarities of feature and expression, as well the nature of the ridge and the position of the lower teeth to the same. If the face be thin and the ridge and lower teeth admit, the centrals may with advantage be so set as to form an obtuse angle, with the laterals slightly inclining to lap over or—as we sometimes find them in the study of the natural teeth—the two centrals on a plane with the laterals, a trifle back, leaving the canines a little prominent. Still another peculiarity with similar features is for the centrals to slightly droop, or to be a sixteenth of an inch longer than the laterals, lengthening at the cuspids. This latter arrangement—taking a side view of the antagonizing edge—gives a curve similar to that of the natural teeth, called a semilunar curve. But should the face be round and full, the laterals should be set up in line with the centrals and be more regular. If the natural antagonism is for the teeth to strike plumb and the patient be advanced in life, the teeth on the cutting edge should be slightly ground off to give them an abraded appearance. The oral arch arranged, the bicuspids and molars will naturally find their own position. In grinding the teeth to the plate, it is very necessary to have the joints come fairly together, and every part of the tooth that is possible to be in contact with the plate; this will obviate accidents in soldering and prevent warping, and leave no space for the accumulation of the food. To accomplish this paint should be used to indicate the part to be ground off. The teeth being arranged and articulated, they should be removed one at a time and backed; and as each backing is finished the tooth should be replaced; this will keep them in position in the arch. In finishing the backing and before the tooth is replaced, it should be marked at the base of the tooth as a guide in fitting it to the plate. The backings made and the teeth in position, the piece may be removed from the model and imbedded for soldering. This is done by investing the plate and teeth in plaster and asbestos, equal parts, about half an inch under and around the teeth and plate, and extending an eighth of an inch over the cutting and grinding edges. After sitting the wax or cement—that held the teeth in position—can be removed, and the pins and plate well cleaned; the plate should then be well scraped where the backings meet it, so that solder will attach itself firmly. When care is taken at this stage of the work, the cracking of the teeth and warping of the plate may, in a great measure, be prevented, especially in fitting the backings so as to have them in actual contact with the plate; also in bending or splitting the pins, no more force should be uged than is necessary to hold the backing to the tooth. The borax should be ground to the consistency of cream, and the lining, pins and tooth well coated and then fastened to the tooth by pressing the pins gently together with the pliers. This done, solder—previously coated with borax—may be placed on the pins and backing where it is to be united to the plate, using no more solder than is necessary to unite the same. A piece as long as the width of backing and about 1-16 of an inch wide — if the backing is in contact with the plate—will be found sufficient with a piece about the 1-16 of an inch square to each pin. This, if evenly flowed, will give little trouble in finishing.

*To Solder.* I use a small hand furnace, and igniting well the charcoal, place the piece to be soldered on it and build pieces of coal over it. Let the furnace have an upright position without draft—no more than the perforated bottom gives, and allow the heat to come up gradually until the borax is melted; then flow the solder with the mouth blow pipe, and afterwards cool the piece gradually. After it is cold, remove the investment and place the piece in the sulphuric acid bath to remove the oxide and borax, when it is ready for finishing. In finishing, to remove the excess of solder and pins use small burr wheels attached to the lathe, these are about the size of a three cent piece with file cut edges, and do the work of an hour by hand in a few minutes; then with a stiff brush wheel and oil and tripoli powder remove all traces of file marks. After washing, the plate is burnished and applied to a fine brush wheel—this time using whiting and alcohol, when with a cotton buff wheel the finishing touch is given with rouge and alcohol, and when washed will be found to have a very high polish.

Nothing now remains except to adjust the piece to the mouth.

---

### ARTICLE III.

### *Recent Views on Digestion.*

